# Organics Challenge Ammonia as Hydrogen Carriers

**DOI:** 10.1021/acscentsci.2c01272

**Published:** 2022-11-01

**Authors:** Alexander H. Tullo

For 8 months in 2020, the Japanese engineering firm Chiyoda conducted an ambitious demonstration. At a natural gas processing plant in Brunei, toluene molecules were saturated with hydrogen to form methylcyclohexane (MCH). Container ships carried tanks of the liquid 5,000 km to Kawasaki, Japan. There, a Chiyoda-designed plant heated the MCH to more than 350 °C over a proprietary catalyst, dehydrogenating it back into toluene. A local refiner, TOA Oil, used the resulting hydrogen to power a gas turbine; Chiyoda shipped the spent toluene back to Brunei to start the process all over again.

Chiyoda and its Japanese partners involved in the project ferried 102 metric tons (t) of hydrogen to Japan during this demonstration. The company proved the concept again in 2022 using larger chemical tankers.

“This was also a success, so that means we are ready for commercialization,” says Osamu Ikeda, a business development manager at Chiyoda. The firm envisions establishing a vast network connecting hydrogen-producing locales like the Middle East, North America, and Australia with consumers in Japan, Singapore, western Europe, and other places with an appetite for clean energy.

The idea of liquid organic hydrogen carriers (LOHCs) like the MCH-toluene system is hardly new. It has been obvious to chemists for decades that they could load cyclic and heterocyclic compounds with hydrogen for storage and transport.

In 1986, scientists from Italy’s Institute CNR for the Transformation and Storage of Energy wrote a prescient piece in Precious Metals Review about the potential of “MTH”—their term for the MCH-toluene-hydrogen cycle. The authors “hoped that in time, as fossil fuels are increasingly depleted, the needs of the hydrogen economy will match the prospects offered by the MTH process, like a cure in search of a disease.”

The time these scientists foresaw may have arrived, albeit with climate change rather than fossil fuel scarcity as the ailment. Governments and industry are looking for safe and economical ways to ship green hydrogen over great distances. Companies such as Chiyoda and the Japanese refiner Eneos see their moment and are clearing final technical hurdles for MCH. A German start-up, Hydrogenious LOHC Technologies, is scaling up a benzyl toluene–based system. Compounds like *N*-ethylcarbazole might also emerge as commercial hydrogen carriers.

**Figure:**
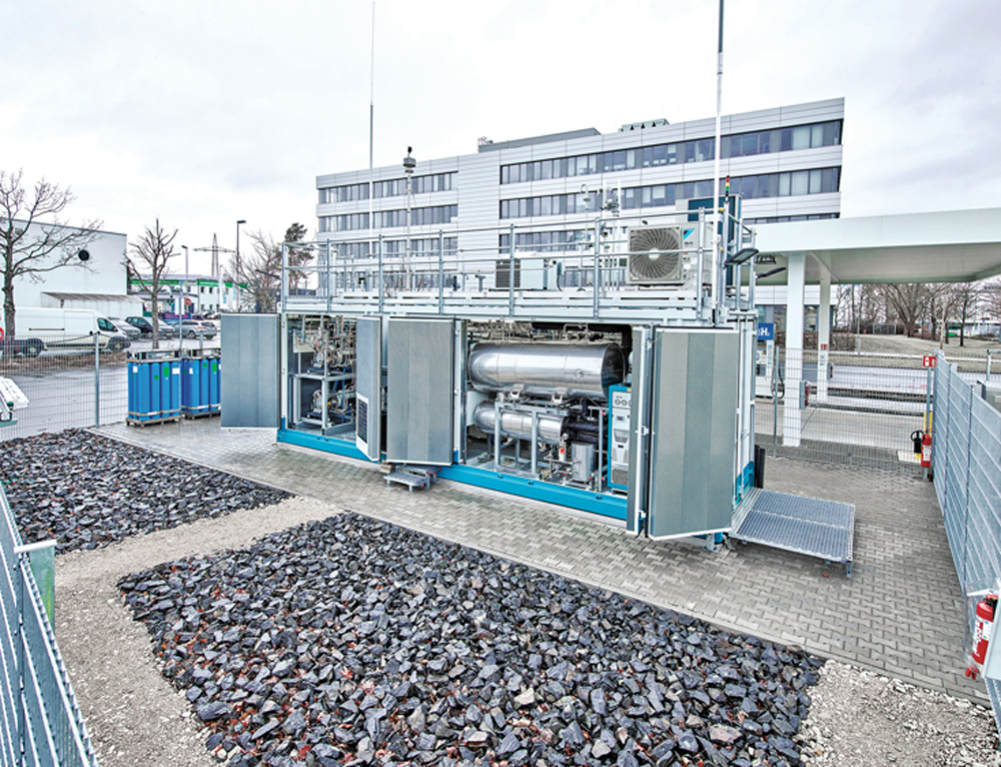
A hydrogen fueling station in Erlangen, Germany, uses a system from Hydrogenious LOHC Technologies that is based on benzyl toluene. Credit: Hydrogenious LOHC Technologies

But LOHCs already have a formidable rival. In recent years, ammonia has attracted most of the attention—and investment—in hydrogen transport.

Air Products is involved in a $5 billion project in Saudi Arabia that will split water with renewable power to make green hydrogen and then ammonia. The firm intends to ship the ammonia around the world to truck and bus hydrogen fueling stations, where it will be cracked back into hydrogen. And fertilizer makers like Nutrien intend to sell low-carbon ammonia to customers that will burn it directly in power plants or in specially designed ship engines.

Ammonia has much to recommend itself. It has been a major industrial chemical for more than a century. Chemical makers know how to produce and ship it in vast quantities.

It also has technical attributes that lend themselves to the hydrogen economy. Scientists from the Tokyo Institute of Technology recently published a paper comparing liquid hydrogen, ammonia, and MCH. All three lose significant amounts of energy when they’re used to transport hydrogen. Liquefying hydrogen isn’t cheap. Ammonia synthesis and decomposition both come with a major energy penalty. And dehydrogenating MCH is a highly endothermic reaction.

According to the paper, ammonia’s total energy efficiency is about 34–37%, while liquid hydrogen’s is 30–33% and MCH’s is only about 25%. Chiyoda engineers dispute the MCH figure and say its efficiency is better. Moreover, ammonia has the highest hydrogen density of the three. MCH, which has a molecular weight overwhelmingly tied up in carbon, has the lowest.

But MCH is easier to handle than ammonia. Ammonia is a gas under standard conditions, and high concentrations of it can be caustic. MCH, in contrast, is a liquid under ambient conditions. “It is suitable for long-term storage and also long-distance transportation because MCH and toluene can be handled similar to gasoline,” Chiyoda’s Ikeda says. “We are able to use existing infrastructure, like the tanks, tankers, pipelines, and lorries.”

And while ammonia has been made commercially for more than a century, cracking it to release hydrogen hasn’t been tested on a large scale, notes Daniel Teichmann, founder and CEO of Hydrogenious. The reaction conditions are harsh—for example, occurring at about 800 °C. And gaseous hydrogen has to be separated from nitrogen and nitrogen oxides, a potentially convoluted step for local hydrogen fueling stations.

“A lot of investment needs to be done in order to have molecular hydrogen coming out of it in the end,” Teichmann says.

He founded Hydrogenious about a decade ago with a blank slate to find the most practical LOHCs. The firm looked at MCH and *N*-ethylcarbazole, among others, but chose to develop benzyl toluene and dibenzyl toluene instead. It recently settled on benzyl toluene because of its lower viscosity at cold temperatures.

Teichmann claims advantages over MCH. Benzyl toluene, used for decades as a heat-transfer fluid that operates over a wide range of temperatures, is even stabler than toluene. It’s “practically nonflammable,” he says. The high stability of benzyl toluene also means that less of it is degraded during hydrogenation—about 0.1% of it per cycle, roughly a tenth the rate of MCH.

Minyoung Yoon, a chemistry professor at Kyungpook National University who has authored studies on LOHCs, sees MCH and benzyl toluene as the leading candidates for commercialization. Other molecules, such as N-ethylcarbazole, are relatively expensive even if they are less energy intensive to dehydrogenate.

**Figure:**
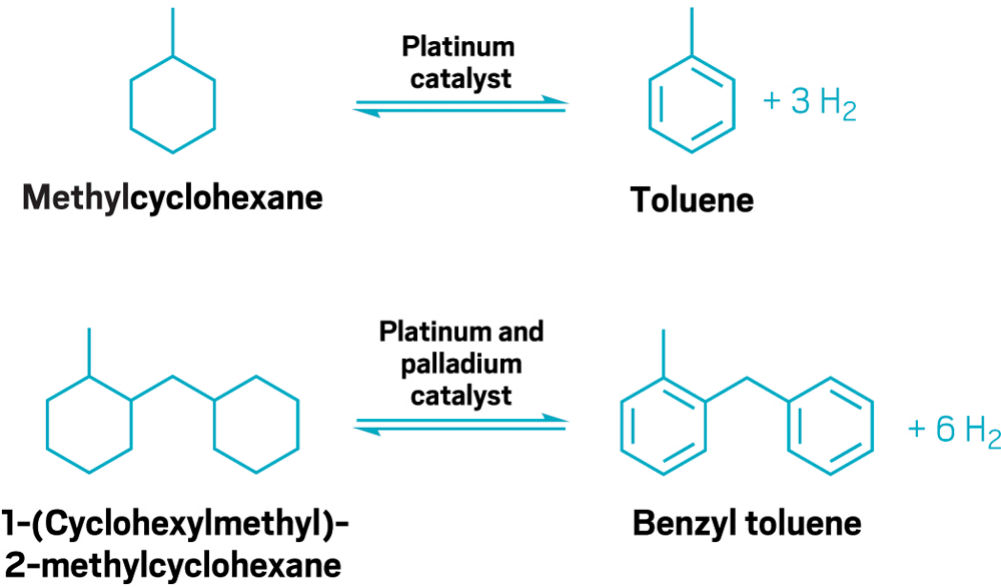
Methylcyclohexane and benzyl toluene are two of the top candidates to be liquid hydrogen carriers. Credit: C&EN

The low boiling point of MCH means that some users will have to clean vapor out of the hydrogen stream before use. But high hydrogen purity might not matter for applications like power plants. “The MCH-toluene system may be suitable for the specific application,” Yoon says in an email.

Hydrogenious is pursuing two main tracks for its technology. It is working on stationary installations at places like refineries, steel plants, and hydrogen fueling stations. To that end, the firm has been collaborating with Clariant on a dehydrogenation catalyst made from platinum on an aluminum oxide carrier. Longer term, Hydrogenious is partnering with the maritime services firm Østensjø Rederi for the development of onboard dehydrogenation systems to power ships, a technology that could eventually be applied to trains, trucks, and buses. “What we really focus on today is the scale,” Teichmann says, “bringing it to multitons per day and ultimately multihundred tons per day of hydrogen capacity.”

For example, Hydrogenious is building a facility in Dormagen, Germany, at the site of one of its investors, Covestro. Using benzyl toluene, it will capture 2,000 t per year of by-product hydrogen from Covestro’s chlorine plant. Hydrogenious also intends to ship up to 10,000 t per year of hydrogen from Sweden to Rotterdam, the Netherlands. It has even bigger plans with Abu Dhabi National Oil Co. (ADNOC) to ship several hundred tons per day out of the United Arab Emirates.

Chiyoda has similar scale-up plans. Now that it has demonstrated its MCH system, the company aims to have commercial installations capable of processing tens of thousands of tons per year of hydrogen by the middle of this decade. It hopes to reach hundreds of thousands of tons by around 2030. Earlier this year, Chiyoda won a share of a grant from the Singapore government to establish a hydrogen supply chain there. It is also participating in a study with the Port of Rotterdam.

Commercialization would not have been possible without the MCH dehydrogenation catalyst, which Chiyoda has been developing since 2002. “The most important issue for the development of this technology is the high stability of the catalyst,” says Fuyuki Yagi, Chiyoda’s director of R&D.

Yagi explains that conventional MCH dehydrogenation catalysts have low activity and low lifetimes—only about several hundred hours of operation—because of coke buildup. The Chiyoda catalyst, which is based on platinum particles on alumina supports, is 100 times as active as other catalysts and can run continuously for over 2 years, Yagi says.

Eneos, the oil refiner, is developing innovative chemistry that might reduce the cost of forming MCH. The firm reacts water and toluene together in an electrochemical cell to produce MCH, circumventing water electrolysis, as well as the hydrogen tanks and handling required for conventional MCH production.

Working with Chiyoda and Queensland University of Technology, Eneos has demonstrated electrolyzers with 2 kW of power. Eneos plans to demonstrate a 150 kW electrolyzer this year and aims for a 5 MW electrolyzer by 2025.

In the meantime, Eneos is rolling out collaborations using conventional MCH synthesis. It launched a study with the Malaysian oil company Petronas to load MCH with hydrogen from Petronas’s petrochemical plants and ship it to Japan. With ADNOC and Mitsui & Co., it plans to ship 50,000 t per year of hydrogen in MCH from Abu Dhabi to Japan.

These companies are betting that even if ammonia gets the starring role in the hydrogen economy, LOHCs will play a supporting one. “We think ammonia is a very important option, but it is not superperfect,” Chiyoda’s Ikeda says. “That is why MCH can also be covered and coexist.”

*Alexander Tullo is a senior correspondent at**Chemical & Engineering News**, the independent news outlet of the American Chemical Society. A version of this story appeared in C&EN.*

